# Pervasive Strong Selection at the Level of Codon Usage Bias in *Drosophila melanogaster*

**DOI:** 10.1534/genetics.119.302542

**Published:** 2019-12-23

**Authors:** Heather E. Machado, David S. Lawrie, Dmitri A. Petrov

**Affiliations:** * Cancer, Ageing, and Somatic Mutation, Wellcome Sanger Institute, Hinxton CB10 1SA, UK; † Department of Ecology and Evolutionary Biology, University of California, Irvine, California 92697-3958; ‡ Department of Biology, Stanford University, California 94305-5020

**Keywords:** codon usage bias, CUB, selection, splicing, synonymous sites, *Drosophila*

## Abstract

Codon usage bias (CUB), where certain codons are used more frequently than expected by chance, is a ubiquitous phenomenon and occurs across the tree of life. The dominant paradigm is that the proportion of preferred codons is set by weak selection. While experimental changes in codon usage have at times shown large phenotypic effects in contrast to this paradigm, genome-wide population genetic estimates have supported the weak selection model. Here we use deep genomic population sequencing of two *Drosophila melanogaster* populations to measure selection on synonymous sites in a way that allowed us to estimate the prevalence of both weak and strong purifying selection. We find that selection in favor of preferred codons ranges from weak (*|N_e_s| ∼* 1) to strong (*|N_e_s| >* 10), with strong selection acting on 10–20% of synonymous sites in preferred codons. While previous studies indicated that selection at synonymous sites could be strong, this is the first study to detect and quantify strong selection specifically at the level of CUB. Further, we find that CUB-associated polymorphism accounts for the majority of strong selection on synonymous sites, with secondary contributions of splicing (selection on alternatively spliced genes, splice junctions, and spliceosome-bound sites) and transcription factor binding. Our findings support a new model of CUB and indicate that the functional importance of CUB, as well as synonymous sites in general, have been underestimated.

THE degeneracy of the genetic code leads to protein-coding mutations that do not affect amino acid composition. Despite this, such synonymous mutations often have consequences for phenotype and fitness. The first evidence of the functionality of synonymous sites came from the discovery of codon usage bias (CUB), where, for a given amino acid, certain codons are used more frequently in a genome than expected by chance (Grantham *et al.* 1981; Ikemura 1981). While the consensus in the field is that CUB is often driven by natural selection, the nature and strength of natural selection acting to maintain CUB is disputed.

The most common explanations for CUB postulate selection on either the rate or the accuracy with which ribosomes translate mRNA to protein (Hershberg and Petrov 2008). The existence of selection at synonymous sites at the level of translation is supported by several key observations. First, the preference toward particular “preferred” codons is consistent across genes within a particular genome suggesting a global, genome-wide process, and not preference for the use of particular codons within specific genes (Grantham *et al.* 1980; Chen *et al.* 2004). Second, optimal codons tend to correspond to more abundant tRNAs, suggesting a functional relationship between translation and CUB (Post *et al.* 1979; Ikemura 1981, 1982; Qian *et al.* 2012). Third, preferred codons are more abundant in highly expressed genes than in the rest of the genome (Gouy and Gautier 1982; Bulmer 1991; Novoa and Ribas de Pouplana 2012), consistent with selection being proportional to mRNA transcript abundance. Finally, constrained amino acid positions tend to contain preferred codons more frequently, suggesting a link between CUB and translational accuracy (*Escherichia coli:* Stoletzki and Eyre-Walker 2007; *Drosophila melanogaster*: Akashi 1994; mammals: Drummond and Wilke 2008). In addition to speed and accuracy, there is evidence that other processes are affected by codon composition, such as cotranslational folding (Pechmann and Frydman 2013), RNA stability (Presnyak *et al.* 2015; Carneiro *et al.* 2019), and transcription (Carlini and Stephan 2003; Newman *et al.* 2016; Zhou *et al.* 2016).

Beyond the level at which selection operates to generate CUB, it is important to consider how strong selection at synonymous sites is likely to be. This question has been addressed with population genetics approaches introduced by the seminal papers of Li and Bulmer (Li 1987; Bulmer 1991). The Li–Bulmer model proposes that the observed, relative proportion of codons can be explained by the balance of mutation, selection (in favor of preferred codons), and random genetic drift. This model assumes a constant selection coefficient per codon or codon preference group, and predicts that the strength of selection in favor of preferred codons should be on the order of the reciprocal of the effective population size (*N_e_s ∼* 1). The predicted weak selection should be detectable as a slight deviation in the site frequency spectrum (SFS). Mutations from preferred to unpreferred codons should reach comparatively lower frequencies in the population than those in the opposite direction. Such deviations have, in fact, been observed in many organisms that show clear CUB (*D. melanogaster*: Zeng and Charlesworth 2009; *Caenorhabditis remanei*: Cutter and Charlesworth 2006; *E. coli*: Sharp *et al.* 2010), supporting the conclusion that selection at synonymous sites is weak but detectable.

While weak selection driving CUB may match the intuition that a synonymous change should not have a large phenotypic effect, there is abundant experimental evidence that this is not always the case. For example, optimizing the codon composition of the viral protein BPV1 increases the heterologous translation of the protein in humans by >1000 fold (Zhou *et al.* 1999). In humans, a change in codon composition in the gene *KRAS*, from rare to common codons, increases KRas protein expression and is associated with tumorigenicity (Lampson *et al.* 2013). In *D. melanogaster*, changing a small number of preferred codons to unpreferred codons in the alcohol dehydrogenase (*Adh*) gene resulted in substantial changes in gene expression and in ethanol tolerance (Carlini and Stephan 2003; Carlini 2004). The authors estimated that such unpreferred codons are subject to *s* > 10^−4^—much larger than the Li–Bulmer predictions of the strength selection strength on synonymous sites. The Bulmer 1991 paper acknowledged this generally as a puzzle in need of solving, as discrepancies between their theoretical predictions of the strength of selection and that of mechanistic models of CUB were already apparent.

We can characterize the selection acting on CUB using population genetic data. One commonly used method of estimating level of selection is to compare the SFS of a putatively selected class of sites to that of a neutral reference. This approach is powerful, as the neutral reference can make the test independent of the demographic history of a population. These and similarly constructed tests have been used to estimate the strength of selection on CUB in *D. melanogaster*, and have typically failed to find any evidence of strong selection on CUB (Singh *et al.* 2007; Zeng and Charlesworth 2009, 2010; Clemente and Vogl 2012a; Campos *et al.* 2013). However, strong purifying selection results in an enrichment of very low allele frequency variants, requiring very deep population sequencing to allow for the detection of its effects in SFS data. In the absence of very deep and accurate population sequencing, an alternative method is to utilize information about the proportion of sites that are polymorphic (polymorphism level). Since both strong purifying selection and a decreased mutation rate can lower the polymorphism level, the selected class of sites would have to be compared with a neutral reference that is matched for mutation rate and levels of linked selection. Thus, the limit of detection for strong selection in the aforementioned studies, which either ignored polymorphism level or did not have a control for it, was set by the lowest allele frequency class in the dataset (set by the number of individuals sampled).

Intriguingly, a study by Lawrie *et al.* (2013) that did incorporate polymorphism level and SFS with the use of matched neutral controls did find evidence of strong purifying selection in synonymous sites, but was unable to correlate this substantial selection with CUB. As the authors did not have enough variants in their study to test the SFS of preferred sites separately, they could not test for weak selection on CUB or distinguish strong selection from lethal CUB-associated mutations.

Here, we test the prevalence of strong purifying selection on CUB in two distinct *D. melanogaster* populations (the DGRP Freeze 2 dataset and a high diversity African population). We accomplish this by comparing the polymorphism level and SFS of fourfold degenerate synonymous sites in preferred and unpreferred codons to that of a short intron neutral reference. The neutral reference is produced by matching each fourfold site to a short intron site that is located within 1 kb and has the same nucleotide at the position of interest and at the 5′ and 3′ neighboring sites. This creates a neutral reference that is subject to the same mutation rate and environment of linked selection as the fourfold sites. We find evidence that there is a distribution of selection strengths on CUB, ranging from weak to strong. Our findings of strong selection on CUB directly conflict with previous models of CUB that predict uniformly weak selection. Further, we find that CUB explains a large proportion of the signal of strong selection on synonymous sites, indicating that the functional effects of CUB have been generally underestimated.

## Materials and Methods

### Sequence data

We used sequence data from two *D. melanogaster* population samples, one from North America (DGRP Freeze 2), consisting of 200 inbred lines (Mackay *et al.* 2012), and one from Africa (Zambia), consisting of 197 haploid embryos (Lack *et al.* 2015), downloaded from the *Drosophila* Genome Nexus (http://www.johnpool.net/genomes.html). To reduce the effect of sequencing and mapping error, for each individual we filtered out all sites with low mapping quality (MAPQ *<* 20). We also filtered out sites within 10 bp of an indel. Since mapping errors are more common in regions around indels, and since introns have a greater number of indels, including these regions could have artificially inflated the short intron polymorphism level. We also eliminated polymorphic sites with more than two alleles (∼1% of polymorphisms).

Per population, we downsampled sites to a uniform coverage of 160 haplotypes, and excluded sites with >160 haplotypes. We considered only the four major autosomal chromosome arms because of systematic differences between *D. melanogaster* autosomes and X chromosomes (Singh *et al.* 2005), and polarized polymorphic sites by parsimony, identifying the ancestral state as the allele found in the *D. simulans* v2 reference genome (Hu *et al.* 2013). We used the *D. melanogaster* reference allele for cases where the ancestry was ambiguous, either because there was no direct *D. simulans* alignment, or because neither allele was present in *D. simulans*. While instances of mispolarization can occur, we note that as the polarization is not used for unfolding spectra, mispolarization has little to no effect on the shape of the SFS (*i.e.*, no exchange of mutations from low-to-high frequency), and, therefore, will have little effect on the maximum-likelihood (ML) estimates. Fourfold degenerate synonymous (4D) sites and intronic regions were identified from Flybase annotations (release 5.5; www.flybase.org). The total number of 4D sites in our two datasets was 1,976,830 for DGRP and 1,862,290 for Zambia. We classified short introns (SI) as introns >86 bp in length, and excluded the first and last 8 bp of each intron, as these regions are known to be under constraint (Haddrill *et al.* 2005; Halligan and Keightley 2006; Clemente and Vogl 2012b). The total number of SI sites was 550,587 for DGRP and 446,462 for Zambia.

We created the SI control dataset by matching each 4D site to a SI site. To control for mutation rate differences between 4D sites and their matched controls, we required each matched SI site to have the same ancestral allele and the same neighboring nucleotides (3 bp context) as the 4D site. We matched blind to the direction or strand (*i.e.*, matching with the forward, reverse, reverse complement, or complement SI sequence). To control for the effect of linked selection on the level of 4D polymorphism, we also required each matched SI site to be within 1000 bp of the 4D site, such that SI control would be subject to the same linked selective pressure from nonsynonymous sites as the 4D sites. We found 1000 bp to be a sufficiently small distance, as we found no significant correlation between SI polymorphism and distance between the 4D sites and the matched intron over the range of 0–1000 bp (Supplemental Material, Figure S1).

We produced 200 such matched 4D/SI datasets, each with the same 871,218 DGRP or 754,503 Zambia 4D sites, and an average of 288K SI sites for DGRP and 244K SI sites for Zambia (each SI site is matched to an average of 3 4D sites). Note that we find this decision of SI re-use to be important. For example, if we instead remove 4D sites for which there is not a unique SI match, this results in a skew in the trinucelotide composition, and has a systematic effect on 4D/SI polymorphism levels. Correcting for this skew by further downsampling restores the polymorphism levels but results in too few sites to perform the ML analyses (Figure S2). For confidence interval estimation, each dataset was resampled with replacement. When estimating selection parameters and their confidence intervals on these resampled sets, linkage between 4D sites within these sets would be a violation of the SFS assumptions that posit independence between sites. However, as a consequence of the structure of the data, the above resampling scheme works well as linkage in *D. melanogaster* breaks down across short distances (Feder *et al.* 2012), and the 4D sites used (those with a matching nearby SI site) are sufficiently sparsely populated across the genome.

### Maximum-likelihood estimation of selection parameters from SFS

We employed a variation of the site SFS method described in Lawrie *et al.* (2013). The method uses both SNP density and frequency information of SFS to calculate the distribution of fitness effects (DFE) for a test set of sites given a “neutral” reference—in this case, the DFE for 4D synonymous sites with SI sites as the reference. For the purposes of ML estimation, the spectra are folded. The DFE itself is modeled as a categorical distribution, where the program estimates effective selection coefficients (*γ* = *N_e_s*) and the percentages of sites (*f*) evolving under those selection coefficients for a predetermined number and type of selection categories. This has the advantage of not assuming a particular distribution shape, such as gamma or lognormal, but comes at the cost of additional free parameters per additional categories. For example, a three category model that has a neutral class (*f*_0_) + a weak selection class (*f_W_*, 0 *> γ_W_ > −*10) + a strong selection class (*f_S_*, *−*10 *> γ_S_ > −inf*) requires four free parameters to fully describe it (*f*_0_ = 1 *− f_W_ − f_S_*, *γ*_0_ = 0). The method also estimates the per site effective mutation rate, *θ* (4*N_e_μ*), for the SI spectra. While estimating parameters on SFS, the effective population size, *N_e_*, is held constant, allowing the other parameters to be fit to the data relative to that *N_e_*.

Demography, linked selection, and other forces affecting both 4D and SI sites, can skew the spectra and bias the estimation of the above DFE parameters. To compensate, we used frequency-dependent correction factors, *α_x_*, which adjusts the probability of seeing a site with a SNP at frequency of *x* in the sample − *p*(*x|*model) (Eyre-Walker *et al.* 2006). The likelihood (*λ*) of the SFS under the model’s framework is shown below:

(1) $$\;\lambda_{full}(SFS_{4D},SFS_{SI}|\theta ,\vec{\gamma },\vec{f},\vec{\alpha })=\lambda (SFS_{4D}|\theta ,\vec{\gamma },\vec{f},\vec{\alpha })\times \lambda (SFS_{SI}|\theta ,\vec{\alpha })$$

(2) $$\;\lambda (SFS_{4D}|\theta ,\vec{\gamma },\vec{f},\vec{\alpha })=p{\left(0|\theta ,\vec{\gamma },\vec{f},\vec{\alpha },L,N_{s}\right)}^{k_{0}}\overset{\frac{1}{2}}{\underset{x=\frac{1}{N_{s}}}{\prod }}{\left(\alpha_{x}p(x|\theta ,\vec{\gamma },\vec{f},L,N_{s})\right)}^{k_{x}}$$

${where:\;\alpha }_{\frac{1}{N_{s}}}∶=1;\;{\;N}_{s}∶=\#\;of\;sample\;frequencies;{\;k}_{x}∶=\#\;of\;sites\;at\;frequency\;x;\;L∶=\sum_{x}k_{x}$

(3) $$p(0|\theta ,\vec{\gamma },\vec{f},\vec{\alpha },L,N_{s})=1-\overset{\frac{1}{2}}{\underset{x=\frac{1}{N_{s}}}{\sum }}\alpha_{x}p(x|\theta ,\vec{\gamma },\vec{f},L,N_{s})$$

(4) $$p(x|\theta ,\vec{\gamma },\vec{f},L,N_{s})=\underset{c}{\sum }\left[p_{c}(x|N_{e},\mu ,\gamma_{c},L_{c},N_{s})+p_{c}(\left(1-x\right)|N_{e},\mu ,\gamma_{c},L_{c},N_{s})\right]$$

$where:\;L_{c}∶=f_{c}L;\;p\left((|0.5|\theta ,\vec{\gamma },\vec{f},L,N_{s}\right)∶=\sum_{c}p_{c}\left(0.5|N_{e},\mu ,\gamma_{c},L_{c},N_{s}\right)$

As in Lawrie *et al.* 2013, the likelihood of the model to explain the full data set is the product of the likelihoods of the model explaining the 4D and SI data independently (Equation 1). The model itself is defined by *θ*, the array of *γ* coefficients for each selection class (*c*), the array of *f* fraction of sites in each class, and the array of *α* demographic-correction parameters. For each set of sites, the likelihood is calculated using the multinomial distribution where the probability of seeing a site at a given sample frequency, *p*(*x|.*..), in the population is modified by an *α* parameter and exponentiated by the actual number of sites at frequency *x* (Equation 2). In the analyses where all sites, including monomorphic, are included in the analysis the probability of seeing a site in the monomorphic, “zero”, class is 1—probability of seeing a polymorphism (Equation 3). Equation 4 shows the folded probability of finding a mutation at frequency *x* in the sample over all selection classes. The likelihood equations for the short intron sites are the same as Equations 2–4 above except that the model is one of pure neutrality (*f*_0_ = 1, *γ*_0_ = 0), but it shares the same *θ* and *α* parameters.

A major difference from Lawrie *et al.* (2013) is in calculating *p_c_*(*x|.*..), the probability of seeing a mutation at frequency *x* in the finite sample for a given selection class *c*. Lawrie *et al.* (2013) used an approximation that resulted in the effects of finite sampling being canceled out. This approximation allowed for the faster calculation of probabilities and worked well for estimating the number of sites in each selection category and for estimating the strength of weak selection regimes. However, for strong purifying selection, the approximation causes our method to underestimate the strength of selection by ∼10–30%. In our current study, we calculated the probability *p_c_*(*x|.*..), accounting for the effects of finite sampling using the binomial distribution:

(5) $$p_{c}(x|N_{e},\mu ,\gamma_{c},L_{c},N_{s})=\overset{\frac{2N_{e}-1}{2N_{e}}}{\underset{z=\frac{1}{2N_{e}}}{\sum }}g_{c}(z|\mu ,\gamma_{c},L_{c})\times binompdf(N_{s}x;N_{s},z)$$

(6) $$g_{c}(z|\mu ,\gamma_{c},L_{c})=\left\{\begin{array}{cc} 2\mu L_{c}\times \frac{1-e^{-4\gamma_{c}(1-z)}}{z(1-z)(1-e^{-4\gamma_{c}})} & \gamma_{c}\neq 0\\ 2\mu L_{c}/z\;\;\;\;\;\;\;\;\;\;\;\;\;\;\;\;\;\;\;\;\;\;\;\;\;\;\;\;\;\;\;\;\;\;\;\;\; & \gamma_{c}=0 \end{array}\right.$$

In Equation 5, *binompdf* (*k*; *N_s_*, *z*) is the binomial probability of choosing *k* out of *N_s_* derived alleles at a frequency *z* in the population. Equation 6 is the standard Wright-Fisher model for calculating, *g_c_*(*z| …*), the probability of finding a mutation at a frequency *z* in a diploid population with codominant alleles where the fitness of the derived, mutant allele is 1+2*s* the fitness of the ancestral allele (Wright 1938).

### Model parameters for demography and linkage

All inferences were made with an *N_e_* of 2000. This parameter was chosen to be big enough for the binomial sampling assumptions to hold, while still being computationally tractable. Assuming a constant effective population size is convenient for generating theoretical spectra, but deviations of the putatively neutral SI SFS from the theoretical neutral SFS are expected to exist due to an organism’s demographic history, as well as factors such as linkage to sites under selection. While our paired-bootstrap method endeavors to ensure a similar overall level of linked selection affecting 4D and SI spectra, any residual skew from linkage must still be corrected for when estimating selection parameters. To account for this deviation of the SI SFS from the theoretical neutral, we performed a ML fit of offsets (*α* values) for each allele frequency bin. Frequency-dependent correction factors produce a fidelity in re-estimating statistics from simulated spectra comparable to commonly used, computationally tractable, demographic models (Tataru *et al.* 2017). The frequency spectra were divided, according to a power law, into six separate allele frequency bins with the same α_x_ within each bin (Table S1). For speed, the demographic parameters were first estimated on the SI data alone before the rest of the model was estimated on the entirety of the data set [tests did not reveal any significant differences between estimating the *α* parameters on the whole data set, *vs.* on the SI data set (not shown)].

### Model parameters for selection

We tested five different ML models: (1) neutral, (2) neutral + lethal, (3) neutral + 1 selection coefficient, (4) neutral + selection + lethal, and (5) neutral + 2 selection coefficients. For ML estimation without polymorphism level data, see Supplemental Material, Text S2. The neutral + 2 selection coefficients model requires a parameter that is the boundary condition between weak and strong selection classes. We tested a broad range of boundary conditions and found *N_e_s* = *−*10 to permit all ML peaks to be reached for differentiating strong and weak selection. Thus, selection categories with *N_e_s* < *−*10 classify strong purifying selection. The lethal class is defined purely by a drop in polymorphism density, with no concomitant excess of rare alleles—essentially infinitely strong purifying selection. In practice, it represents all selection effects stronger than what can be observed with the SFS, and is indistinguishable from a drop in mutation-rate relative to the neutral control. This latter property is why it is important to differentiate strong, but finite, purifying selection from lethality.

The ML program requires seed values for selection strength, selection proportion, lethal proportion, and theta. To identify the highest likelihood model, we used the Matlab function fminsearch, an implementation of the Nelder–Mead simplex method (Nelder and Mead 1965), with multiple seed values to bolster the chance of finding the global maximum (see Table S2 for seed values). To determine the best fit model, we performed a chi-squared likelihood ratio test of the ML scores. Since the chi-squared test is approximate for determining significance in a composite-likelihood (Coffman *et al.* 2016), we also use confidence intervals. To calculate 95% confidence intervals, we performed a rank bootstrap, sampling with replacement each of the 200 matched 4D and SI datasets, performing our ML estimate of selection and using the 5th and the 195th rank values for each ML score, proportion of selection, and strength of selection.

### Model interpretation

Our program takes a nonparametric approach to modeling selection and demography. In the case of demography, the frequency-dependent correction factors skew the mutation–selection equilibrium SFS to fit the data. While in the true, unknown, evolutionary history of the sites being studied, the amount of effective selection on deleterious mutations fluctuates over time due to demographic events and linkage, the inferred *N_e_s* values returned by our method are the mutation–selection balance equivalent—a single, constant *N_e_s* value that captures the average amount of effective selection experienced by the typical deleterious mutation in these sites.

For selection, there is some unknown distribution of selection coefficients over the sites, referred to as the Distribution of Fitness Effects (DFE), which we have represented with either one or two selection masses. ML inference using this categorical model estimates what the proportion of sites, and how strong the selection in each category has to be in order to produce the same overall SFS as the true, unknown DFE. It has been shown in the literature that the use of such point estimates to represent the DFE provides an unbiased description for a range of real underlying DFEs (Eyre-Walker and Keightley 2007; Kousathanas and Keightley 2013). A simple example of the behavior of our inference model can be found in Figure S3. Here, we performed the ML inference using only two selection categories (neutral and deleterious) on simulated data with true underlying DFEs with three selection categories: neutral, weakly deleterious, and strongly deleterious. The lone inferred deleterious selection category is forced to represent the total amount of selection in the system with varying degrees of success for the different scenarios, but, in all cases, the interpretability of a single non-neutral selection category can be problematic. In contrast to parametric DFE inference, these inferred selection categories should be viewed as descriptors of the true underlying DFE, rather than a model of the DFE itself.

### Power analysis and model validation

In order to assess our power in differentiating strong selection from a lethal class or 4D/SI mutational differences, we performed power analyses of our ML method of selection estimation. We did this by creating theoretical spectra for a range of selection strengths and proportions with theta values reflecting those we found in our ML inferences for the DGRP (0.01) and Zambia (0.035) populations. We then estimated selection for these spectra using a theoretical neutral reference with the same theta value and number of sites. We performed a chi-squared likelihood ratio test with one degree of freedom comparing the 2-category selection model (neutral + one selection class) with the neutral + lethal model. This analysis demonstrates how an increasing number of SNPs, increasing polymorphism level (*e.g.*, larger theta), and a greater proportion of sites under selection increase our power to distinguish strong selection from lethality/mutational differences (Figure S4, A and B).

We then tested the power and biases of using a categorical DFE in combination with frequency-dependent correction factors to account for demography when demography was complex. We simulated sites with two selection categories evolving both under mutation–selection equilibrium and under a bottleneck/growth demographic scenario which had been estimated for Zambia (Table 2 in Singh *et al.* 2013: third codon model) and then attempted to re-infer the simulation parameters. These Wright-Fisher simulations were run using a modified version of the program GO Fish (Lawrie 2017). In Figure S4, C–G, we find that, overall, the estimation of the percentage of sites in the non-neutral selection category is robust to demographic effects. One exception is when the selected sites are very weakly deleterious, in which case the proportion of such sites is underestimated in the bottleneck/growth demographic model. Likewise, our estimates of the strength of purifying selection become increasingly conservative (underestimated) for simulations with stronger selection against deleterious mutations. Further, we lose power to distinguish finite strong purifying selection from lethality quicker than when sites are evolving under mutation–selection equilibrium. We also ran three selection category simulations with similar results (not shown). The primary cause for the increased difficulty in making these estimations for the bottleneck/growth demography model with respect to mutation–selection equilibrium is that mutations in different selection regimes respond to the same nonequilibrium demography differently, causing different skews in the SFS for each. The frequency-dependent correction factors meanwhile infer a global skew that modifies the spectra of all selection regimes the same way.

Most of the potential biases mentioned above are small, and all of them are conservative. Supposing, for example, that the demographic model used above is indeed an accurate reflection of the evolutionary history of the Zambian population: as compared to what we report in Table 1 for Zambia preferred 4D sites, the true strength of selection in the strong categories may actually be underestimated; meanwhile, the confidence intervals for the proportion of sites in the weak selection category are already quite wide, so a small underestimation of their tally does not change much.

**Table 1:** Nested maximum-likelihood models tested for the Zambia and DGRP datasets

Population	Dataset	Model	Prop. S1	-Ns S1	Prop. S2	-Ns S2	Delta LL	P
Zambia	Full	n	—	—	—	—	—	—
		n + l	0.09 (0.09–0.10)	Inf	—	—	255	1*10*^−^*^111^
		n + s	0.12 (0.11–0.14)	26 (14–44)	—	—	303	3*10*^−^*^22^
		n + s + l	0.10 (0.06–0.90)	14 (5–33)	0.03 (0–0.06)	Inf	307*	3*10*^−^*^3^
		n + s + s	0.06 (0–0.158)	7 (0.1–10)	0.07 (0.03–0.13)	81 (18-Inf)	306	1
DGRP	Full	n	—	—	—	—	—	—
		n + l	0.13 (0.12–0.14)	Inf	—	—	239	1*10*^−^*^104^
		n + s	0.14 (0.12–0.15)	Inf (75-Inf)	—	—	241*	0.04
		n + s + l	0.11 (0–0.87)	166 (0-Inf)	0.03 (0–0.14)	Inf	241	1
		n + s + s	0 (0–0.01)	0.2 (0.1–6)	0.14 (0.12–0.15)	Inf (76-Inf)	241	1
Zambia	Preferred	n	—	—	—	—	—	—
		n + l	0.18 (0.16–0.19)	Inf	—	—	446	2*10*^−^*^194^
		n + s	0.30 (0.27–0.33)	7 (4–11)	—	—	669	9*10*^−^*^99^
		n + s + l	0.23 (0.18–0.28)	3 (1–6)	0.07 (0.03–0.10)	Inf	691	1*10*^−^*^11^
		n + s + s	0.82 (0.19–0.86)	0.3 (0.2–2)	0.16 (0.10–0.20)	29 (15–75)	699*	9*10*^−^*^5^
DGRP	Preferred	n	—	—	—	—	—	—
		n + l	0.25 (0.24–0.26)	Inf	—	—	512	2*10*^−^*^223^
		n + s	0.29 (0.27–0.31)	29 (12–57)	—	—	574	1*10*^−^*^28^
		n + s + l	0.14 (0.08–0.21)	3 (1–10)	0.15 (0.09–0.19)	Inf	597*	2*10*^−^*^11^
		n + s + s	0.15 (0.11–0.79)	2 (0.1–5)	0.19 (0.14–0.24)	116 (52-Inf)	598	0.1

n: neutral; l: lethal; s: selection. Maximum-likelihood parameter estimates per model (median of 200 sets of matched 4D and SI sites). Values in parentheses are 95% bootstrap confidence intervals. Model comparison was performed with *chi*^2^ goodness of fit test. *Best fit model with *P* < 0.05. The delta log-likelihood (Delta LL) is with respect to the neutral model, while the *P* value is with respect to the nested model above.

To further validate our methodology, we checked for consistency between real Zambia and DGRP data sets and the expected spectra generated from our model using another SFS inference method, DFE-alpha (Eyre-Walker and Keightley 2009). We find a good fit between DFE-alpha parameter estimates for the observed spectra and our model-generated spectra (see Supplemental Material, Text S1).

### Polymorphism ratio estimate of strong selection

In order to make a precise estimate of purifying selection using our SFS-based ML method, we require a large number of sites (*>* 100K). When we have few sites, we can use alternative methods for estimating purifying selection. One proxy for the amount of strong purifying selection is the depletion of polymorphism in a selected class compared with a neutral class. We quantified this depletion as the “polymorphism ratio”, which is a variation of the classic statistic, *P*_N_*/P*_S_, for comparing the density of neutral and selected segregating sites. However, since polymorphism can at times be *greater* at 4D sites given certain selection scenarios, we opted to use a statistic that was symmetric with regard to the 4D or SI polymorphism depletion and use the log of this ratio: log(*P*_SI_*/P*_4D_), where *P*_SI_ and *P*_4D_ are counts of the number of polymorphic sites after the matching of SI and 4D sites.

Because strong purifying selection is expected to remove polymorphism from the dataset, we tested whether this simple polymorphism log-ratio statistic, which quantifies the depletion of polymorphism in 4D sites relative to SI sites, can capture the number of 4D sites under strong purifying selection. As we show using simulated data (Figure S5A), this statistic provides a largely unbiased estimate of strong purifying selection, even when the number of sites is significantly less than what is required for the ML estimates. Note that there is only a roughly one-to-one relationship between polymorphism ratio and the amount of strong purifying selection, and this varies with the strength of selection (Figure S5A). Weak selection does affect the polymorphism ratio as well; however, it does so to a much lesser extent. In our datasets, the correlation between our ML estimates and polymorphism ratio measures most closely resembles the pattern predicted by strong purifying selection (Figure S5B). We therefore use this metric as a proxy for the proportion of sites under strong purifying selection for subsets of sites, specifically when there are too few sites to perform ML inference. It is important to remember that the linear relationship between polymorphism ratio and proportion of sites under strong purifying selection may not hold for all datasets. While we demonstrate that this relationship does generally apply to our data, the linear relationship, and, thus, this analysis may not be applicable to other datasets (*e.g.*, where there are large differences in selection strengths across datasets).

### Identification of putatively functional regions

#### Codon usage bias:

We calculated the relative synonymous codon usage (RSCU) for each codon as the observed frequency of a codon in the dataset divided by the expected usage if all four codons were used equally (0.25) (Sharp and Li 1986). We classified each 4D site as being in a preferred (highest RSCU for the amino acid) or unpreferred codon (lowest three RSCUs for the amino acid). The amino acids and their respective preferred codons are as follows: alanine GCC, glycine GGC, leucine CTG, proline CCC, threonine ACC, and valine GTG. For polymorphic 4D sites, we used the ancestral allele to designate the codon and preferred state. We identified a total of 850,973 (366,458 with SI controls) and 794,471 (312,523 with SI controls) 4D sites in preferred codons for DGRP and Zambia, respectively. When analyzing sets of 4D sites, all sites with the same ancestral codon are grouped together, regardless of any derived polymorphism.

We additionally measured the polymorphism ratio for individual 4D mutations (*e.g.*, CCC→CCA), and examined the relationship between the resulting RSCU change and polymorphism ratio. In order to appropriately calculate the polymorphism ratio for each codon change, we matched 4D sites to all SI sites with the same possible states. For example, for the class of 4D sites of an ancestral “CCC” proline codon and a derived “CCA” proline codon, we matched the 4D proline “C” monomorphic sites and “C→A” polymorphic sites to either SI “C” monomorphic sites or “C→A” polymorphic sites (or the complement), as well as matching for distance and mutational context.

#### Transcription factor binding sites:

We used modEncode chromatin immunoprecipitation sequencing (ChIP-seq) experiments to assess the contribution of transcription factor binding (TFB) sites to the signal of purifying selection on synonymous sites (http://intermine.modencode.org). This dataset represents 25 experiments, testing 15 transcription factor targets (antibodies: odg-GFP, anti-trem, Sin3A-RC, Su(var)3-9, KW4- PCL-D2, KW3-D-D2, KW3-Trl-D2, bon (GP37), HP1 antibody (ab24726), HP1-Covance, KW4Hr39-D1, KW3-Kr-D2, KW3-CG8478-D1, KW3-hkb-D1, KNI-D2,KW3-Trl-D2; modENCODE submissions 3229, 3230, 3232, 3234, 3237, 3238, 3239, 3240, 3241, 3242, 3243, 3245, 3390, 3391, 3392, 3393, 3394, 3395, 3396, 3398, 3399, 3400, 3401, 3402, 3403). We consider a “transcription factor bound region” to be any region with evidence for TFB in any of the noncontrol experiments (minimum binding score: 50). We identified a total of 294,703 (129,611 with SI controls) and 289726 (118,463 with SI controls) transcription factor (TF) bound 4D sites for DGRP and Zambia, respectively.

#### Spliceosome binding:

We used modEncode RNA immunoprecipitation sequencing (RIP-seq) experiments targeting putative spliceosome proteins to assess the contribution of spliceosome binding to the signal of purifying selection (http://intermine.modencode.org). The experiments tested for RNA-protein binding of a total of 30 putative splicing proteins. We considered a region to be bound if it had a binding score of 5 or greater in any of the experiments. This left a total of 321,290 (153,037 with SI controls) and 316,740 (137,967 with SI controls) spliceosome-bound 4D sites for DGRP and Zambia, respectively.

#### Alternative splicing:

We distinguished between genes with and without alternative splicing using the analysis in Brown *et al.* (2014). We considered any gene with more than one transcript as alternatively spliced. We found a total of 1,196,063 (635,814 with SI controls) and 1,136,535 (556,673 with SI controls) 4D sites in alternatively spliced genes for DGRP and Zambia, respectively.

#### Splice junctions:

We used the splice junctions identified by Brooks *et al.* (2015). We found 18,410 (13,447 with SI controls) and 17,528 (11,920 with SI controls) 4D sites in splice junctions for DGRP and Zambia, respectively.

#### Ribosomal occupancy:

We estimated ribosomal occupancy using the ribosomal profiling experiments conducted by Dunn *et al.* (2013). We first normalized each pooled experiment file (GEO accession GSE49197) by dividing the number of counts in each region by the total number of counts across regions. All regions with zero counts for either the footprinting or expression experiments were excluded. We estimated ribosomal occupancy by dividing the normalized ribosomal footprint values by the normalized expression values (for each DNA strand separately). The top and bottom 1 percentile of ribosomal occupancy scores were omitted from downstream analysis, leaving translational efficiency scores for 1,391,585 4D sites. We defined high ribosomal occupancy as the top 25% of values (represented by 116,645 and 104,168 SI-matched 4D sites for DGRP and Zambia, respectively).

#### Frequency of preferred codons:

We calculated the frequency of preferred codons (FOP) per gene. As before, preferred codons were defined as the most frequent codon for a given amino acid. The FOP was calculated with our 4D datasets, such that codons that did not appear in our datasets (*e.g.*, those without 4D sites) did not contribute to the FOP calculation. Sites were classified as being in genes with either low (bottom quartile), medium (middle two quartiles), or high (top quartile) FOP. The average proportion of preferred codons for sites in low, medium, and high FOP genes was 28, 42, and 54%, respectively.

#### Estimating the number of sites under strong purifying selection in functional class subsets:

As most of the functional class subsets have too few sites to perform the ML estimate of purifying selection, we use the polymorphism ratio to calculate a rough estimate the number of sites under strong purifying selection in each subset. We estimate the number of sites under strong purifying selection by calculating the increase in polymorphism ratio in a functional class above the background polymorphism ratio (the polymorphism ratio for sites in a functional class minus the polymorphism ratio for the remaining sites), multiplied by the total number of sites in the functional class (pre-SI matching).

### Conservation scores

We calculated the level of conservation of each 4D site across a 10-species *Drosophila* phylogeny. We excluded *D. melanogaster* from the phylogenetic analysis in order to avoid a confounding effect of *D. melanogaster* polymorphism on both polymorphism ratio and phyloP score. The PRANK multiple sequence alignments of the 10 species (*D. simulans*, *D. sechellia*, *D. yakuba*, *D. erecta*, *D. ananassae*, *D. pseudoobscura*, *D. persimilis*, *D. virilis*, *D. mojavensis*, *D. grimshawi*) were generously provided by Dr. Sandeep Venkataram. We calculated the probability of conservation for each 4D site using the *phyloP* function of the PHAST software (method=“likelihood ratio test”) (Cooper *et al.* 2005).

### Recombination rate

We used *D. melanogaster* recombination rate estimates from Comeron *et al.* (2012). The recombination rate used is the mean across 100-kb windows, and is expressed in cM/Mb/female meiosis. We split the 4D sites into three recombination rate categories: low [bottom third: (0–1.08) cM/Mb/meiosis], medium [middle third: (1.08–2.71) cM/Mb/meiosis], and high [top third: (2.71–14.8) cM/Mb/meiosis].

### Data availability

The authors state that all data necessary for confirming the conclusions presented in the article are represented fully within the article. All data used in this study are publicly available, and are referenced in *Materials and Methods*. The Supplemental Materials file, including Supplemental Text, Figures and Tables, is available at FigShare. Matlab code for the ML estimation of selection is available on Github: https://github.com/DL42/SFS_DFE_categorical. The modified version of the GO Fish program used to generate the Wright-Fisher simulations is also available on Github: https://github.com/DL42/3P_custom_synonymous. Supplemental material is available at figshare: https://doi.org/10.25386/genetics.11316794.

## Results

### Sequence data and neutral controls

We identified all 4D synonymous sites and putatively neutral SI sites in two datasets, one of an African (Zambia) and one of a North American (DGRP Freeze (2) *D. melanogaster* population (see *Materials and Methods*). We used short introns as our neutral reference, as *D. melanogaster* short introns have been previously found to be under minimal selective constraint (Haddrill *et al.* 2005; Parsch *et al.* 2010; Clemente and Vogl 2012b). We matched 4D sites to SI sites based on ancestral nucleotide, mutational context, and location (within 1000 bp). As the number of 4D sites was greater than the number of SI sites, we allowed SI sites to be matched to multiple 4D sites. We confirmed that our results are robust to this matching strategy by also performing an analysis matching only one 4D site per SI site, and found our results to be qualitatively unchanged (but with decreased power, Figure S2). We performed the 4D/SI matching 200 separate times, producing 200 SI control sets.

### Many synonymous sites in preferred codons are under strong selection

In order to detect the presence of purifying selection on synonymous sites, we compared the synonymous 4D SFS and polymorphism level (the proportion of polymorphic sites) to that of the matched SI controls. Purifying selection will affect the shape of the SFS and the polymorphism level in a number of ways. First, purifying selection in general removes genetic variation from a population, resulting in a decrease in the polymorphism level. The effect of purifying selection on the shape of the SFS is a function of the strength of selection. Weak purifying selection (*N_e_s > −*10) decreases the density of the SFS at intermediate allele frequencies, and enriches low frequency variants. Strong purifying selection (*N_e_s < −*10) results in an enrichment of very low allele frequency variants, making a skew in the SFS detectable only when a large number of individuals have been sampled. Very strong selection (and lethal mutations: *N_e_s* = *−Inf*) will not affect the shape of the SFS, and will result primarily in a decrease in the polymorphism level.

#### Reduced 4D polymorphism relative to SI polymorphism:

**Figure 1:**
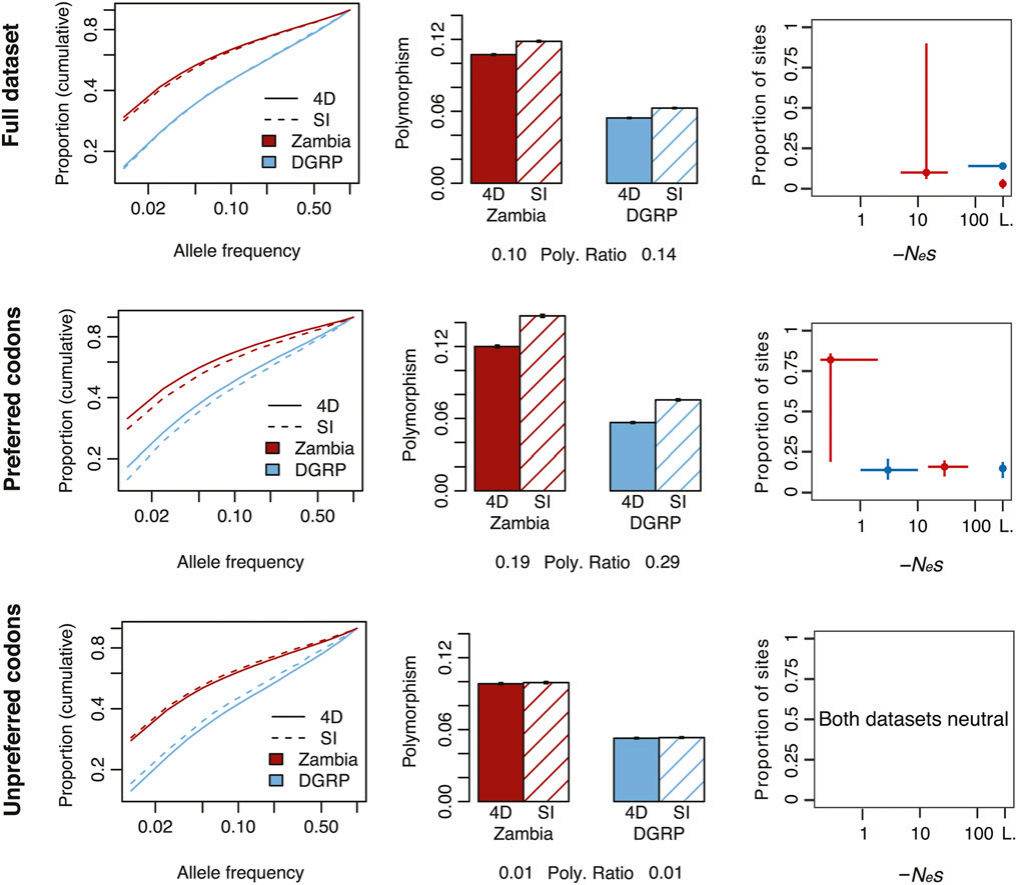
Polymorphism and selection estimates differ by CUB group. SFS (cumulative), polymorphism ratio, and best-fit selection estimates for fourfold synonymous (4D) and matched short intron control (SI) sites for the full dataset (top), for preferred codons (middle), and for unpreferred codons (bottom). L: lethal.

The decrease in 4D polymorphism compared with the SI controls can be expressed as the “polymorphism ratio”, defined as the natural logarithm of the SI polymorphism to 4D polymorphism ratio. We find this statistic to correlate well with the proportion of sites under strong purifying selection estimated by our ML method (Figure S5; see *Materials and Methods* for discussion). A positive polymorphism ratio is due to decreased 4D polymorphism (relative to SI polymorphism) and can be indicative of strong purifying selection. We found a reduction in 4D polymorphism in both the Zambia and DGRP datasets (polymorphism ratio = 0.10 and 0.14, for Zambia and DGRP, respectively; Figure 1). We found an even greater reduction in 4D polymorphism for preferred codons (polymorphism ratio = 0.19 and 0.29 for Zambia and DGRP, respectively), and almost no reduction in 4D polymorphism in unpreferred codons (polymorphism ratio = 0.01 for Zambia and DGRP), suggesting that preferred codons are under more strong purifying selection than unpreferred codons.

#### ML estimates of purifying selection:

The strong reduction in 4D polymorphism is suggestive of strong purifying selection operating on 4D sites; however, this measure is (1) underpowered in detecting weak selection, and (2) does not provide robust quantitation of selection strength or proportion of sites under selection. We used a ML estimation to quantify the strength and extent of purifying selection (see *Materials and Methods* for discussion), testing five different selection models: (1) neutral, (2) neutral + lethal, (3) neutral + 1 selection coefficient, (4) neutral + 1 selection coefficient + lethal, and (5) neutral + 1 weak selection coefficient + 1 strong selection coefficient (Table 1 and Figure 1 and see *Materials and Methods*). For the full Zambia dataset, the best fit model is the neutral + selection + lethal model, which inferred strong selection (10% at *N_e_s* = *−*14) with a small lethal class. The lack of a weak selection estimate for the full dataset is consistent with previous findings that the signal of weak selection is too low for detection when including all sites. Similarly, for the full DGRP dataset, our best-fit selection estimate also inferred a strong selection class; however, the bootstrap 95% confidence interval includes lethality, indicating that the neutral + 1 selection coefficient model was not significantly better than the neutral + lethal model (13% lethal). The DGRP dataset has half the number of polymorphisms as the Zambia dataset (*∼*47K in DGRP), and, thus, the strongest selection that can routinely be distinguished from lethality is *N_e_s* ∼ *−*60 (for power analyses, see Figure S4). With fewer sites, the test lacks power in the DGRP data, but its results are consistent with the presence of strong purifying selection in 4D sites as found using Zambia data.

When we narrow the focus to 4D sites in preferred codons, a class of sites putatively enriched for purifying selection, the ML analysis is able to discriminate more features of the distribution of selection coefficients operating on 4D sites. The best-fit model for Zambia preferred codons is the neutral + 2 selection coefficient model (82% at *N_e_s* = *−*0.3, 16% at *N_e_s* = *−*29), indicating a range of weak and strong selection coefficients acting at the preferred sites. The 95% confidence interval for the proportion of sites in the weak selection category is large (19–86%), but, importantly, both the weak and strong purifying selection classes are distinguishable from each other, from neutrality, and from lethality. The corresponding model for DGRP likewise supports a range of selection effects in preferred sites (15% at *N_e_s* = *−*2, 19% at *N_e_s* = *−*116). Estimating selection in the DGRP dataset is, again, hampered by lower power as this model is not a significantly better fit than the neutral + selection + lethal model, with the 95% confidence intervals overlapping lethality for the strong selection class. Though lacking power, these DGRP results provide independent support for the findings in Zambia of a wide range of selection strengths affecting mutations in preferred 4D sites in *D. melanogaster*.

4D sites in unpreferred codons show a corresponding lack of purifying selection, with the neutral model having the best fit to the data. This is consistent with the low polymorphism ratios for these datasets (0.01 for both Zambia and DGRP, Figure 1). The enrichment of sites under selection in the set of preferred codons and the lack of selection found in the set of unpreferred codons indicates that selection on CUB is a major component of the total amount of purifying selection on synonymous sites, and that the identification of both a weak and a strong selection class for preferred codons indicates that selection on CUB may not be limited to weak selection.

### Phylogenetic conservation scores support finding of strong selection on CUB

**Figure 2:**
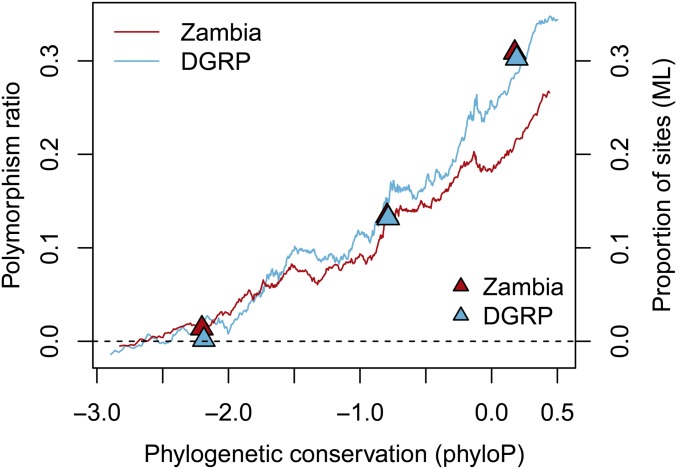
Phylogenetic conservation correlates with selection estimates. Correlation between the phyloP conservation score across a *Drosophila* phylogeny, and the proportion of sites under selection, as estimated by the polymorphism ratio (lines) and the maximum-likelihood (ML) method (triangles). Dark red: Zambia, light blue: DGRP. The polymorphism ratio was estimated in sliding windows of 100K SNPs. The ML estimates were made for three groups: the lowest quartile, the middle two quartiles, and the highest quartile of phyloP scores. ML estimates are plotted against the median phyloP score for each group.

If our ML and polymorphism ratio estimates truly do reflect selection levels, we might also expect our estimates to correlate well with signatures of long-term selection, such as phylogenetic conservation. We calculated phyloP phylogenetic conservation scores across a 10-species *Drosophila* phylogeny (excluding *D. melanogaster*). The phyloP score measures the extent of conservation or divergence per site, with positive values representing conservation and negative values representing divergence. We asked if there was a correlation between the proportion of sites we identified to be under purifying selection and the level of phylogenetic conservation. We found a strong correlation between polymorphism ratio and phyloP conservation score of 4D sites (Zambia: *R*^2^ = 0.96, *P <* 2*10*^−^*^16^; DGRP: *R*^2^ = 0.94, *P <* 2*10*^−^*^16^; Figure 2). We also performed ML estimates of the proportion of sites under selection for 4D sites in low (lower quartile), medium (middle two quartiles), or high (upper quartile) phyloP scores. Again, we observe the same relationship of increasing purifying selection with increasing conservation (Figure 2). The correlation of phylogenetic conservation with our estimates of purifying selection supports the relevance of our estimates to long-term constraint.

### Level of preference for a codon predicts proportion of sites under strong selection

**Figure 3:**
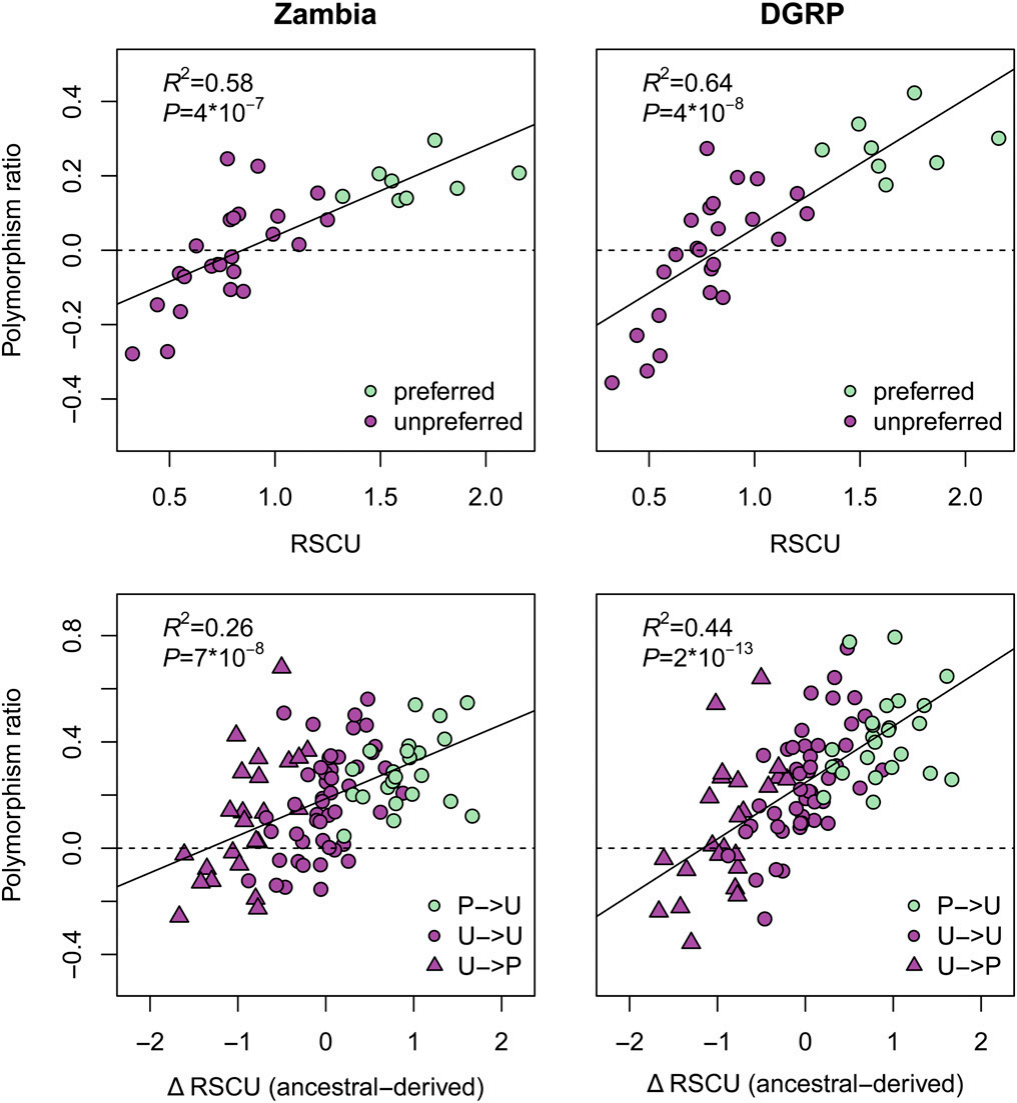
Level of CUB correlates with strong selection measurements. Top: Polymorphism ratio for each codon as a function of the level of bias for the codon (relative synonymous codon usage: RSCU) (median over 200 matched controls). Bottom: Polymorphism ratio for each ancestral/derived codon pair as a function of the change in RSCU.

Our findings suggested that a substantial proportion of synonymous sites in preferred codons were under strong purifying selection. Since the biased usage of codons actually exists on a continuum, rather than binary designations of “preferred” and “unpreferred”, we next asked whether or not the *level* of biased usage (for a particular codon) correlates with the amount of strong selection observed. We used the relative synonymous codon usage (RSCU) as a measure of the level of codon preference (Sharp and Li 1986). We measured RSCU for each 4D codon and compared that to the polymorphism ratio. We found a strong positive relationship between RSCU and polymorphism ratio (Zambia: *R*^2^ = 0.56, *P* = 4*10*^−^*^7^; DGRP: *R*^2^ = 0.63, *P* = 4*10*^−^*^8^; Figure 3).

We next asked if the change in RSCU, from ancestral to derived, correlated with polymorphism ratio. We hypothesized that mutations to a less preferred state (positive RSCU change) would show evidence for strong purifying selection (positive polymorphism ratio), whereas mutations to a more preferred state (negative RSCU change) would be positively selected for, and have an increased level of 4D polymorphism relative to the SI control (negative polymorphism ratio). We found a strong, positive relationship between RSCU change and polymorphism ratio, with negative polymorphism ratios for strongly preferred derived mutations on unpreferred ancestral codons (Figure 3). Under the assumption that SI sites are neutral, negative polymorphism ratios (*i.e.*, greater levels of polymorphism at 4D than SI sites) can result from particular mutation and selection regimes (McVean and Charlesworth 1999; Lawrie *et al.* 2011), such as positive selection on 4D sites increasing 4D polymorphism. Our results support the hypothesis of purifying selection on the strongest unpreferred changes and positive selection on the strongest preferred changes.

### More selection on synonymous sites due to CUB than due to other processes

**Figure 4:**
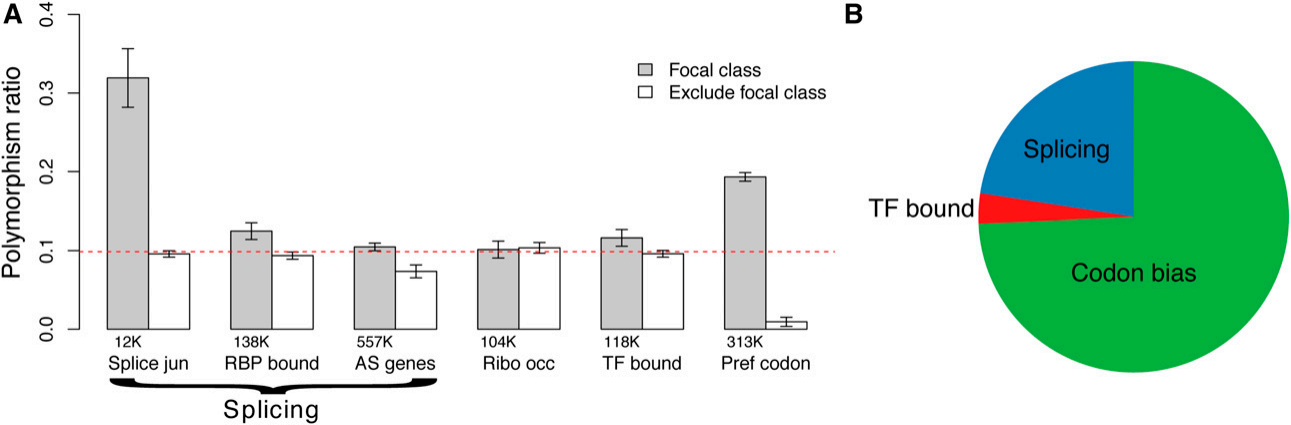
(A) Extent of strong purifying selection as measured by the polymorphism ratio for each class of site (gray) and the dataset excluding the focal class sites (white) (Zambia dataset). The number of sites in a focal class is listed below the corresponding bar. The red dashed line is the polymorphism ratio for the full dataset. Error bars represent 2 SE. (B) Relative proportion of synonymous sites under strong purifying selection due to splicing, CUB, or transcription factor (TF) binding.

Several processes other than those related to CUB have also been hypothesized to act on synonymous sites. In order to assess the relative importance of various processes driving the observed selection on synonymous sites, we tested several putatively functional classes of sites for enrichment of purifying selection. In addition to preferred codons, we tested TF bound regions, alternatively spliced genes, RNA binding protein (RBP) bound regions, splice junctions, and high ribosomal occupancy regions. We calculated the polymorphism ratio for each functional class, and compared it to that of the set of sites excluding the functional class—the latter providing the background level. We found a significantly elevated (above background) polymorphism ratio not only for preferred codons but also for alternatively spliced genes, spliceosome bound regions, splice junctions, and TF bound regions in both the Zambia and the DGRP populations (Figure 4 and Figure S8).

We then estimated the relative contribution of each functional class to the signal of strong purifying selection (see *Materials and Methods*). We combined the three splicing-related classes (alternatively spliced genes, spliceosome bound regions, and splice junctions), leaving three general groups putatively under purifying selection: CUB, splicing, and transcription factor binding. In the Zambia dataset, we estimated there to be 150K sites under strong purifying selection associated with CUB, 38K with splicing, and 4K with transcription factor binding (Figure 4). The DGRP dataset showed similar trends: 217K sites under strong purifying selection associated with CUB, 100K with splicing, and 13K with transcription factor binding. In summary, we found that CUB explained the greatest number of 4D sites under purifying selection, representing ∼2–4 times as many sites as splicing and transcription factor binding combined.

We also measured the polymorphism ratio for the sites least likely to be under selection. We excluded the two largest contributors to selection on synonymous sites: preferred codons and alternatively spliced genes. The set of unpreferred codons in nonalternatively spliced genes consisted of 137K sites in Zambia and 158K sites in DGRP, and represented the 4D sites least likely to be under strong selection. Interestingly, we found that this set of 4D sites had more polymorphism than their SI matched control set (negative polymorphism ratio), indicating greater purifying selection in short introns and/or the presence of positive selection on these 4D sites.

### Recombination rate does not influence strong selection on CUB

**Figure 5:**
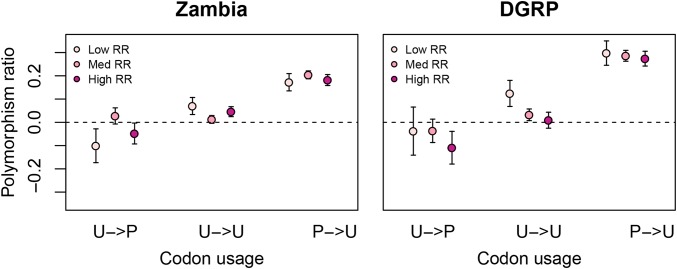
Polymorphism ratio by codon preference and recombination rate (RR). RR groups are classified into low (lowest third), medium (middle third), and high (top third). U: unpreferred; P: preferred. Arrows separate ancestral (pre-arrow) and derived (postarrow) states. Error bars are 2 SE.

Previous studies have found mixed evidence of a correlation between recombination rate and CUB (Kliman and Hey 1993; Marais *et al.* 2001; Singh *et al.* 2005; Campos *et al.* 2012, 2013). We therefore tested for increased levels of purifying selection on preferred 4D sites as a function of recombination rate. While there is a greater proportion of preferred codons in high recombination rate regions (42.1% and 42.6% for Zambia and DGRP, respectively) than in low recombination rate regions (40.1% and 41.2% for Zambia and DGRP, respectively; both *chi*^2^ *P <* 10*^−^*^15^), we found no evidence of increased strong purifying selection on preferred codons in high recombination rate regions compared with those in low recombination rate regions (Figure 5), nor any general increase in selection with recombination rate (Figure S7).

### Features of strong selection on CUB by gene type or genic location

Preferred codons may be under greater purifying selection in some genes than in others. We asked if a greater proportion of preferred codons were under strong purifying selection in genes with high CUB compared to genes with low CUB. One measure of the amount CUB in a gene is the FOP. We calculated FOP per gene and asked if, as expected, there was a stronger signal of purifying selection on 4D sites in genes with higher FOP. We found a trend toward a larger polymorphism ratio for 4D sites in high FOP genes (Zambia: 0.103; DGRP: 0.150) compared with low FOP genes (Zambia: 0.087; DGRP: 0.131), albeit the trend was not significant (Zambia: *t*-test *P* = 0.19; DGRP: *t*-test *P* = 0.26; Figure S6).

**Figure 6:**
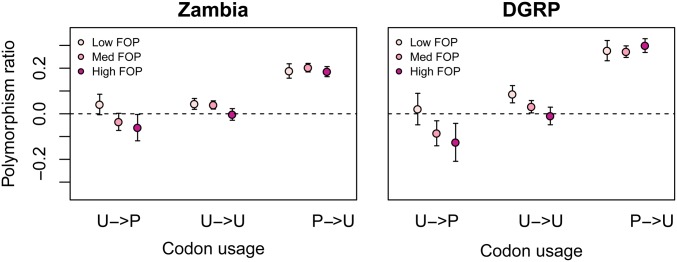
Polymorphism ratio by class of codon preference and the frequency of preferred codons (FOP). FOP groups are classified into low (lowest quartile), medium (middle two quartiles), and high (top quartile). U: unpreferred; P: preferred. Arrows separate ancestral (pre-arrow) and derived (postarrow) states. Error bars are 2 SE.

We then evaluated the patterns of CUB-associated polymorphism by grouping 4D sites into three categories: preferred, unpreferred with mutations to another unpreferred state, unpreferred with mutations to the preferred state. We found no trend of polymorphism ratio verses FOP for preferred codons, indicating that a similar proportion of preferred codons were under strong selection in genes with low overall biased codon usage compared with genes with high bias and consequently, that a larger *number* of preferred codons in high FOP genes are subject to strong selection (Figure 6).

Interestingly, we found a pattern of negative polymorphism ratios for unpreferred codons specifically in high FOP genes, which was particularly pronounced for sites that were ancestrally unpreferred with derived preferred mutations. This pattern was much stronger in high FOP genes than low FOP genes (Zambia: *t*-test *P* = 0.02; DGRP: *t*-test *P* = 3*10*^−^*^5^). This is important for the interpretation of Figure S6, as these negative polymorphism ratios at unpreferred codons in high FOP genes lead to lower overall polymorphism ratios than would be expected given the larger number of preferred codons subject to strong selection in such genes (Figure S6). These patterns are generally consistent with stronger positive selection in favor of preferred codons in high FOP genes (Jackson *et al.* 2017).

**Figure 7:**
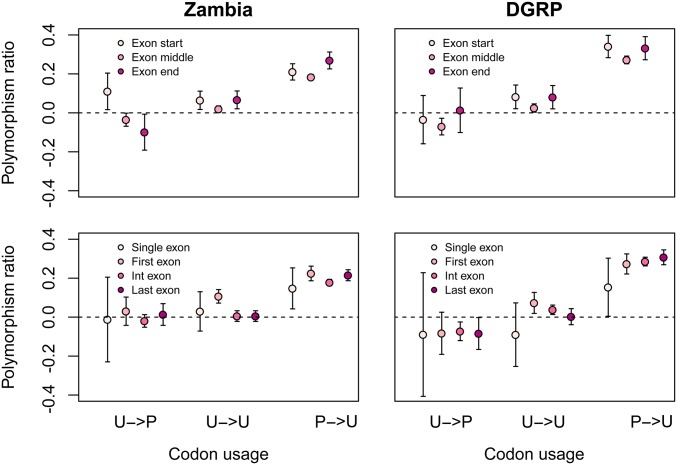
Polymorphism ratio by codon preference and exonic position. Polymorphism ratio by class of codon preference and the position along the exon (top) or the exon position along the gene (bottom). U: unpreferred; P: preferred. Arrows separate ancestral (pre-arrow) and derived (postarrow) states. Error bars are 2 SE.

CUB has also been shown to vary depending on the location in the gene (Plotkin and Kudla 2011). We first asked if preferred codons vary in the amount of strong purifying selection that they are under as a function of the location in the exon. We measured the polymorphism ratio for each class of codon preference at the start (first quartile) of an exon, the middle of an exon (second and third quartile), or the end of an exon (fourth quartile). In preferred codons, we found a trend toward increased polymorphism ratio at the start and the end of exons, compared with the middle of the exons (Figure 7; *t*-test start *>* middle: Zambia *P* = 0.1, DGRP *P* = 0.01; *t*-test end *>* middle: Zambia *P* = 8*10*^−^*^5^, DGRP *P* = 0.03). However, this pattern was also observed in unpreferred codons (*t*-test start *>* middle: Zambia *P* = 0.05, DGRP *P* = 0.04; *t*-test end *>* middle: Zambia *P* = 0.06, DGRP *P* = 0.5), indicating that this effect may be unrelated to CUB. Alternatively, this could be a result of purifying selection on synonymous sites important for splicing. We next assessed polymorphism ratio as a function of the exon position along the gene (either first exon, last exon, intermediate exons, or exons of single-exon genes). No consistent patterns were observed with location of the exon (Figure 7).

## Discussion

### Strong and weak purifying selection on CUB

We find evidence that selection on CUB is not limited to weak selection, and find that 10–20% of 4D sites in preferred codons are under strong purifying selection. Our study builds on methodology developed in Lawrie *et al.* (2013), recapitulating their major result of strong purifying selection on synonymous sites, and extending the analysis to identify functional associations. We were able to gain a finer view by (1) use of multiple, deeply sampled datasets, (2) ancestral polarization of alleles, and (3) strict filtering of sites with low quality or near indels to reduce noise. Although Lawrie and colleagues found evidence of strong selection on 4D sites, the underlying processes examined could not account for this signal. While our finding of strong purifying selection on 4D sites is consistent with the findings of Lawrie *et al.* (2013), our study finds that CUB accounts for the majority of this selection, and, in conjunction with splicing and TFB, can fully account for the decreased levels of 4D polymorphism. In addition to finding that many 4D sites are subject to strong selection, we also find evidence that a substantial proportion of 4D sites are under weak purifying selection on CUB, which is consistent with the signal of weak selection previously observed in *D. melanogaster* (Zeng and Charlesworth 2009, 2010; Campos *et al.* 2013).

For methodological reasons, many previous methods identified only weak selection on CUB. Strong purifying selection is not detectable with methods that use only the polymorphic SFS, without sufficiently high depth of population sequencing (Zeng and Charlesworth 2009, 2010; Campos *et al.* 2013) or methods that incorporate polymorphism level, but assume a DFE that can provide misleading biological inferences if the assumed distribution does not match the true DFE, *e.g.*, the gamma distribution in Eyre-Walker and Keightley (2007), Andolfatto *et al.* (2011) and Kousathanas and Keightley (2013). Other inference methods that incorporate polymorphism level, but without making use of a neutral reference, would also have difficulty detecting strong purifying selection (Zeng and Charlesworth 2009, 2010; Campos *et al.* 2013). In our analysis, we use the polymorphism level and SFS to make point estimates of selection strengths, which is robust to a range of real underlying DFEs (Eyre-Walker and Keightley 2007; Kousathanas and Keightley 2013). This allows us to detect selection occurring at both the weak and the strong range of selection coefficients, such as in the set of Zambia preferred codons, where we detect peaks of selection coefficients at *N_e_s* = *−*0.3 as well as at *N_e_s* = *−*29. Thus, our method is able to infer a wide range of selection strengths operating in 4D synonymous sites.

### Polymorphism ratio correlates with the level of CUB per codon and per gene

Since we control for mutation rate and local determinants of polymorphism such as linked selection and recombination, we find that the polymorphism ratio of the SI to 4D sites can be a good proxy for the proportion of sites under strong purifying selection, as seen in simulations as well as empirically in the relationship between polymorphism ratio and both the ML estimates of selection and the level of phylogenetic conservation (Figure 2 and Figure S5). Further, the estimated proportion of sites under strong selection is highly correlated with the extent of CUB, as measured by the relative synonymous codon usage (RSCU) (Figure 3). The change in RSCU from ancestral to derived also correlates with the proportion of sites under strong selection. These results further support our conclusion of strong purifying selection on CUB.

We also notice negative polymorphism ratios in the RSCU analysis, where we find strongly negative polymorphism ratios for codons that we would expect to be under the greatest amount of positive selection, *i.e.*, codons with highly unpreferred ancestral states and highly preferred derived mutations. Positive selection could be driving an influx of mutations that alter 4D sites from generally less-fit unpreferred states to generally more-fit preferred states. The negative polymorphism ratios are also consistent with excess purifying selection on the control SI sites relative to the tested 4D sites.

It is well established that certain genes, particularly those with high expression, tend to have a greater proportion of preferred codons (Gouy and Gautier 1982; Bulmer 1991; Novoa and Ribas de Pouplana 2012). We measured the polymorphism ratio for sites in genes of low, medium, and high FOP. From this analysis, we have three major findings: (1) the proportion of preferred codons under strong purifying selection is relatively constant across genes (Figure 6), (2) there is evidence for increased positive selection for derived preferred mutations in high FOP genes (Figure 6 and Jackson *et al.* 2017), and (3) the contribution of excess 4D polymorphism from these derived preferred mutations in high FOP genes is an example of how the polymorphism ratio measure of strong purifying selection can be dampened by positive selection. To more fully articulate the third point, polymorphism ratio in high FOP genes is the combination of two competing processes: more ancestral preferred codons increasing the polymorphism ratio, and more positive selection for derived preferred codons reducing the polymorphism ratio. The opposing actions result in no significant increase in overall polymorphism ratio from low to high FOP genes (Figure S6), despite the expectation that a larger number of preferred codons should produce higher estimates of selection in high FOP genes (Jackson *et al.* 2017).

### Selection on other functional classes

We find splicing to be the second-most important process underlying purifying selection on synonymous sites. We tested three classes of sites putatively enriched for selection due to splicing: alternatively spliced genes, spliceosome-bound regions, and splice junctions. Of these three classes, although alternatively spliced genes explain the greatest amount of selection on synonymous sites (*∼*90K sites), owing to the large number of sites in alternatively spliced genes, we find that splice junctions have the greatest proportion of sites under selection (*∼*45% under strong purifying selection), followed by spliceosome-bound regions. Splicing is known to be a critical function for proper development and function of an organism. Similar research in humans has likewise found evidence of widespread, strong evolutionary constraint affecting 4D sites in exonic splice enhancers (Savisaar and Hurst 2018).

There is also evidence for strong selection on TF bound 4D sites. We estimate that *∼*3K 4D sites are under strong selection due to TFB. To identify TF bound sites, we used ChIP-seq experiments targeted at 16 different TFs. With a larger breadth of TFB data, 4D sites in TF-bound regions may prove to be under a greater amount of selection than we can detect here.

We find that CUB, splicing, and TFB are sufficient to explain the polymorphism differences between 4D and SI control sites, indicating that these processes also explain the bulk of strong purifying selection acting on synonymous sites. However, it is important to note that our measures are only correlative with the functional class being tested, such that we cannot say that these processes directly underlie the selection. In addition, there are likely multiple other processes acting on synonymous variants that we have not included. Other processes that have been shown, or hypothesized, to act on 4D sites include transcriptional regulation (Newman *et al.* 2016), protein folding (Rodriguez *et al.* 2018), and RNA transcript stability (Presnyak *et al.* 2015; Carneiro *et al.* 2019) and secondary structure (Gebert *et al.* 2019). Given the explanatory power of our results, we suggest that these other processes are either less affected by synonymous variation or that they are correlated with the processes already tested.

### Controlling for linked selection and mutation rate

One caveat to our polymorphism level based method of estimating selection is that multiple processes can reduce the observed level of polymorphism of a site. These include linked selection, low recombination rate, a reduced mutation rate or selection on the site itself. In order to isolate the effects of selection on 4D sites, we controlled the distance between matched 4D and SI sites so that, on average, the sites in the 4D and SI site frequency spectra were experiencing the same rate of recombination and mutation as well as comparable levels of linked selection. We found that, with an increasing distance of up to 1000 bp from the focal 4D site to its SI control, there was no systematic change in polymorphism in the SI control, indicating that, overall, the matched controls were under a sufficiently similar amount of linked selection (Figure S1). In order to account for mutational differences, we added a further requirement that matched controls have the same 3 bp mutational context as the 4D sites. In *Drosophila*, there is a significant effect of 3 bp context on mutation rate (Sharp and Agrawal 2016). For polymorphic sites we used the ancestral allele for matching (polarized from *D. simulans*), providing a more appropriate match than if we had not polarized by ancestral state. Since we match locally (*<* 1000 bp), 4D sites and their matched SI controls will also be subject to the same local mutation and recombination rate effects. Further, these results should not be confounded by biased gene conversion as this phenomenon appears to be absent in *D. melanogaster* (Robinson *et al.* 2014; Jackson *et al.* 2017), and the local 4D/SI matching would correct for it even if it were active. Finally, we observe that our estimates of purifying selection in polymorphism correlate both with long-term signals of purifying selection in divergence (Figure 2), and with the putative functionality of a class of sites (Figure 4), such as preferred codons, splice junctions, RBP bound regions, and alternatively spliced genes, supporting the claim that our results reflect the action of selection, rather than being artifactual.

### Potential biases in selection estimates

Our estimates of purifying selection on 4D sites may be conservative, underestimating the true amount of selection on 4D sites. This could be the case if there was any constraint on the SI controls, or if there was positive selection on the 4D sites themselves. There were two methodological decisions that may have contributed to constraint in short introns. Both Halligan and Keightley (2006) and Parsch *et al.* (2010) found that short introns (*<*65 bp and *<*120 bp, respectively) have the least constraint on bases 8–30. As we included a larger portion of the intron, it is possible that we have also included SI sites under a greater amount of conservation. We also excluded regions surrounding indels (10 bp on either side) in order to reduce false polymorphisms due to mismapping. This more strongly affects short introns (as they are more permissive to indels than coding regions) and will select for more conserved SI regions. Purifying selection acting on the control SI sites can result in a negative polymorphism ratio, as can positive selection acting on 4D sites. As previously discussed, we actually do observe a negative polymorphism ratio in certain data subsets. Regardless of whether the explanation for a negative polymorphism ratio is purifying selection on control SI sites or positive selection on 4D sites, both dampen our ability to detect purifying selection.

Our estimates of purifying selection can also be biased as the result of demographic forces. For example, a simulated demographic scenario for Zambia shows that our ML model fails to completely correct for the bottleneck/growth demographic scenario, and may result in reduced estimates of the proportion of weakly deleterious sites and the strength of strong purifying selection (Figure S4). Additionally, the sensitivity of our ML model to estimate multiple selection categories is dependent on the number of sites under selection. Deeper population sampling and a larger set of sites supporting more selection categories could reveal greater detail about the true, underlying DFE of 4D sites.

### New model of CUB

Our finding that selection on CUB ranges from weak to strong directly contradicts the standard Li–Bulmer model of selection on CUB. The Li–Bulmer model assumes a constant selection coefficient for a codon, and, given the intermediate proportion of preferred codons observed in many species, predicts that selection on CUB is weak (Li 1987; Bulmer 1991). However, the Li–Bulmer model has not always agreed with the data. First, since population sizes vary by several orders of magnitude across species, the selection coefficient would have to vary inversely by several orders of magnitude as well in order to result in the observed intermediate levels of CUB (Hershberg and Petrov 2008). There is no intuitive reason to think that the selection coefficient would be inversely related to the population size, or that it should vary by several orders of magnitude. Second, if selection is weak, there should be more CUB in high recombination rate regions. This prediction comes from the increased effect of Hill-Robertson interference (linked selection) in low recombination rate regions (Felsenstein 1974; Charlesworth and Campos 2014). While there is some evidence for a correlation between CUB and recombination rate in *D. melanogaster* (Kliman and Hey 1993; Campos *et al.* 2012), this is not true for the *D. melanogaster* X chromosome (Singh *et al.* 2005; Campos *et al.* 2013), and the correlations that have been found can be explained by mutation rate variation (Marais *et al.* 2001). Third, though previous studies of polymorphism data have inferred selection on synonymous sites to be weak (in keeping with Li–Bulmer), these patterns of polymorphism have also suggested a more complex selection model among 4D codons than simply preferred *vs.* unpreferred, contravening the model’s standard formulation (Singh *et al.* 2007; Zeng 2010; Clemente and Vogl 2012a). Fourth, there is experimental evidence that changes in one or more synonymous codons can have large phenotypic and fitness effects, suggesting that selection on CUB is not always weak (Zhou *et al.* 1999; Carlini and Stephan 2003; Carlini 2004; Sharon *et al.* 2018; She and Jarosz 2018; Yannai *et al.* 2018). Indeed, experiments in yeast have found large numbers of synonymous mutations with similar fitness and phenotypic effect sizes to missense mutations, often enriched in genes with high CUB, or causing significant changes in the Codon Adaptation Index (Sharon *et al.* 2018; She and Jarosz 2018).

We propose a new model where the strength of selection per codon varies from nonexistent to strong within a gene, with the level of CUB in a gene set primarily by the distribution of selection coefficients across sites. Genes that have high CUB under our model would have more sites subject to strong selection in favor of preferred codons compared to genes with low CUB, as we in fact see in the data. This eliminates the problem of setting the proportion of preferred codons by fine-tuning the strength of selection at all preferred sites to a particular value of *s* ∼1*/N_e_* under the Li–Bulmer model. In addition, under our model, a substantial proportion of preferred codons is subject to such strong purifying selection (*s >>* 1*/N_e_*), that reduction in effective population size by orders of magnitude due either to demographic shifts or modulation in the strength of genetic draft would still not abolish CUB, as many preferred sites would still remain subject to strong selection (*s >* 1*/N_e_*). At the other extreme, a substantial increase in effective population size would not generate complete CUB as many preferred sites may not be subject to purifying selection at all.

If this model is correct, the key question that remains is what determines whether a particular synonymous site is subject to strong, weak, or no selection in favor of preferred codons. Specifically, the sites under very strong selection might play a disproportionately important role by, for example, being essential for cotranslational folding, transcription, RNA stability, translational efficiency, or translational accuracy. This would suggest that the location of such synonymous sites should be largely conserved across species, as we in fact detect to some extent by showing a correlation between polymorphism ratio and phylogenetic constraint in the *Drosophila* genus (Figure 2).

### Conclusion

We find evidence that CUB is under a substantial amount of purifying selection in *D. melanogaster*, and that this is not limited to weak selection. Our finding that there is a distribution of fitness effects for CUB, ranging from weak to strong selection, resolves the contradiction between the intermediate frequencies of preferred codons observed in most species, and the population-size independence of said frequencies. We also reconcile the observations that changes in synonymous codons can have large phenotypic effects, but that genomic methods have identified only weak selection. We suggest that the reasons previous studies did not find evidence for strong selection on CUB are methodological. Our use of a test that includes the polymorphism level, while controlling for mutation rate and linked selection, provides sufficient power for identifying strong purifying selection. While this study was performed in *Drosophila*, the importance of a new model of CUB is general, as both CUB and the assumption of constant weak selection on CUB is widespread. Further, this study underscores the importance of CUB, and of synonymous variation in general, to the fitness of an organism, and opens research directions to further understand this phenomenon.
